# Investigating interactions between macroinvertebrate indices, water quality parameters, and stream quality classifications in a Wisconsin agricultural watershed

**DOI:** 10.1002/jeq2.70122

**Published:** 2025-12-14

**Authors:** Laura M. Bates, Anita M. Thompson, Laxmi R. Prasad

**Affiliations:** ^1^ Biological Systems Engineering University of Wisconsin‐Madison Madison Wisconsin USA; ^2^ Agricultural and Biosystems Engineering North Dakota State University Fargo North Dakota USA

## Abstract

Increasing agricultural and urban land use poses a growing threat to freshwater ecosystems worldwide, contributing to nutrient loading and degradation of surface water quality. Stream restoration, such as riparian vegetation, can help mitigate nonpoint source pollution, yet its ecological outcomes remain variable. Aquatic macroinvertebrates are widely used as bioindicators for water quality due to their sensitivity to environmental change, but their responses to stream impairment and restoration in the context of ongoing land‐use pressures are less well understood. This study investigates associations between water quality parameters, biotic indices, and stream classifications using macroinvertebrate and water quality data collected from stream sites within an intensively managed agricultural watershed in the Midwestern United States during the autumn seasons of 2021 and 2022. Numerical analyses were used for water quality and macroinvertebrate indices, while stream classifications—impairment status, nutrient thresholds, and habitat quality—were analyzed categorically. Nonmetric multidimensional scaling assessed macroinvertebrate correlations with water quality, while Kruskal–Wallis tests identified statistical differences across stream classifications. Findings show that sites with stream improvement projects exhibit higher macroinvertebrate index scores, with strong correlations between higher index scores, higher dissolved oxygen, and lower total phosphorus. These results suggest macroinvertebrates and water quality parameters are effective indicators of stream impairment and habitat quality, though long‐term links to restoration require further study. Findings contribute to a broader understanding of how biological indicators can inform restoration success and impairment conditions in agriculturally impacted watersheds across temperate regions.

## Core Ideas

- Sample sites with stream improvement projects exhibit higher biotic index scores.
- Higher macroinvertebrate index scores were associated with higher dissolved oxygen and lower total phosphorus.
- Ecological indicators can be used to assess stream impairment.
- Macroinvertebrates can be used to inform nutrient reduction strategies in agricultural watersheds.

## Plain Language Summary

This study looked at how farming and city development affect streams in the US Midwest. When land is heavily used, it often adds nutrients, like phosphorus, to water, which can harm streams and the animals that live there. One way to help is by restoring streams, such as planting vegetation along their edges. Scientists use tiny water animals, called macroinvertebrates, to measure stream health because these creatures respond quickly to pollution. We studied seven streams during the fall of 2021 and 2022, checking water quality and the types of macroinvertebrates living there. We also compared streams that had been restored with those that had not. The results showed that streams with restoration projects had more types of healthy macroinvertebrates, higher oxygen levels, and less phosphorus. This means these small animals and water tests can help show how healthy streams are.

**Abbreviations**
BMP: best management practice
DO: dissolved oxygen
NMDS: nonmetric multidimensional scaling
SWIMS: Surface Water Integrated Monitoring System
TKN: total Kjeldahl nitrogen
TN: total nitrogen
TP: total phosphorus
TS: total solids
WDNR: Wisconsin Department of Natural Resources

## 1 INTRODUCTION

Agricultural and urban land use often contributes to the degradation of water quality and ecological integrity in surface waters (Carpenter et al., 1998; Holmes et al., 2016; Konopacky, 2017). Precipitation can flow over agricultural land and nonporous surfaces as runoff, especially in the winter and early spring months (Prasad et al., 2022), and carry pollutants from manure deposits, leaf litter, and urban streets into streams, rivers, and lakes (Carpenter et al., 2018; Justus et al., 2010; Minnesota Pollution Control Agency, 2017). Because they are difficult to measure and regulate, nonpoint sources are a major contributor of water pollution in the United States (Carpenter et al., 1998; Konopacky, 2018, 2017).

Effective stream restoration can help reverse some of the damages caused by land use changes or create a harmonious balance between these changes and ecosystem health (Cahill et al., 2013; Guo, 2013). As examples, Eagle Creek and Joos Valley Creek, located in Wisconsin's Buffalo County, have successfully been restored (impairment status reversed and sediment load reduction goals exceeded) with best management practices (BMPs) and streambank shaping through the Wisconsin Department of Natural Resources (WDNR) Priority Watershed Program (Graczyk et al., 2010). Other examples in Wisconsin include restoring macroinvertebrate and fish communities in Melancthon Creek, Waldo Creek, and Morgan Coulee Creek through BMPs (Villeneuve, 2007; WDNR, 2024). The degree of hydrologic connectivity from the BMP location to the stream might play a key role in whether BMPs are effective at also improving ecological conditions (Pringle, 2003). Stream restoration projects that involve establishing vegetated buffers and riparian vegetation along stream banks are known to improve both water quality and habitat for aquatic biota (Holmes et al., 2016; Kiffney et al., 2003; Muenz et al., 2006), including macroinvertebrate species. Macroinvertebrates make great candidates for biological indicators and the development of biotic index scores because they are sensitive to changes in their environment (Lowe & LaLiberte, 2017; Suter & Cormier, 2014).

Surface waters that do not meet their chemical, physical, or biological water quality criteria or support their designated uses are impaired (Craig & Roberts, 2015; Prellwitz et al., 2022; WDNR, 2022), which requires a plan for improvement by local agencies. Agricultural BMPs, especially stream restoration projects involving riparian vegetation, have the potential to improve stream water quality and even change a stream's impairment status. Understanding how biological communities are affected by these restoration projects can help watershed managers evaluate the efficiency and location of completed restoration projects and help inform local monitoring efforts. Previous studies have assessed the effects of human disturbance and environmental parameters on stream health (Hamid et al., 2020; Wang et al., 2008), general relationships between macroinvertebrates and habitat quality, land use, or environmental parameters in agricultural watersheds (Heatherly et al., 2007; Stone et al., 2005), and post‐construction impacts to water quality and biological integrity (Chen et al., 2009), but few studies quantify direct effects of BMPs on biotic communities at the watershed scale (Graczyk et al., 2010). Though restoration projects have the potential to mitigate negative impacts from external pollutants, there remain questions of how these projects will hold up under climate change conditions, complex ecosystem interactions, and legacy nutrients (Johnson, 2021), as well as lag times between implementation and pollutant reduction.

This challenge is exemplified by nutrient‐impaired freshwater systems such as Green Lake in Wisconsin, where agricultural runoff has led to high phosphorus levels and oxygen depletion. Since the early 1980s, a range of BMPs and restoration projects have been implemented to reduce phosphorus inputs and improve ecosystem health (Fuller et al., 2022; Johnson, 2021; Johnson et al., 2011). However, the effectiveness of these measures varies significantly depending on site‐specific conditions (GLA, 2024). As agricultural pressures intensify globally, compounded by climate change and persistent internal nutrient loads, restoring nutrient‐impaired lakes remains a major challenge (Prellwitz et al., 2022). Reducing external nutrient inputs through watershed management is essential, and demonstrating ecological improvements from restoration efforts can support future investment in long‐term lake protection.

The objective of this study was to investigate interactions between water quality parameters and macroinvertebrates, and how these parameters and macroinvertebrates reflect stream impairment, restoration, and habitat quality in Green Lake's tributaries. This objective was addressed by first assessing overall water quality associations in the Green Lake Watershed using public reports and water quality data. Then, water quality parameters and macroinvertebrate indices were compared between stream impairment status, habitat quality, and where streams have undergone restoration projects. Results from this research can help create metrics for stream restoration project effectiveness and impairment status based on ecological conditions, as well as aid in guiding decisions regarding aggressive nutrient reduction strategies. In addition, this study provides a roadmap for prioritizing stream improvement initiatives, enhancing public data synthesis, and better understanding water quality's impact on biological integrity within agriculturally dominated watersheds. The following research questions are addressed:

1. Does stream water quality reflect overall stream quality (impairment, restoration/improvement, habitat rating) at the time of sampling (summer and autumn)?
2. Do macroinvertebrate indices reflect water quality parameters at the time of sampling (autumn)?
3. Do macroinvertebrate indices reflect stream quality at the time of sampling (autumn)?

## 2 MATERIALS AND METHODS

### 2.1 Study location

Green Lake in Wisconsin (43.81827, −88.99629) was listed by the WDNR as impaired for high phosphorus concentrations and low metalimnetic dissolved oxygen (DO) in 2014. Forty‐one percent of Green Lake's water sources come from runoff and inflow from seven tributaries, 51% from rainfall, and 8% from springs, and over 65% of the drainage area is agricultural (GLA, 2024). Vegetable crops and beef cattle dominate the agricultural land usage of the watershed, which encompasses Green Lake, Fond du Lac, and Winnebago Counties. Streambank and shoreline protection projects, which are intended to mimic or enhance biological practices by adding riparian vegetation to prevent erosion and maintain sufficient stream flows, make up 10% of the implemented BMP projects since 1981 (GLA, 2024).

**Core Ideas**

- Sample sites with stream improvement projects exhibit higher biotic index scores.
- Higher macroinvertebrate index scores were associated with higher dissolved oxygen and lower total phosphorus.
- Ecological indicators can be used to assess stream impairment.
- Macroinvertebrates can be used to inform nutrient reduction strategies in agricultural watersheds.

**FIGURE 1:**
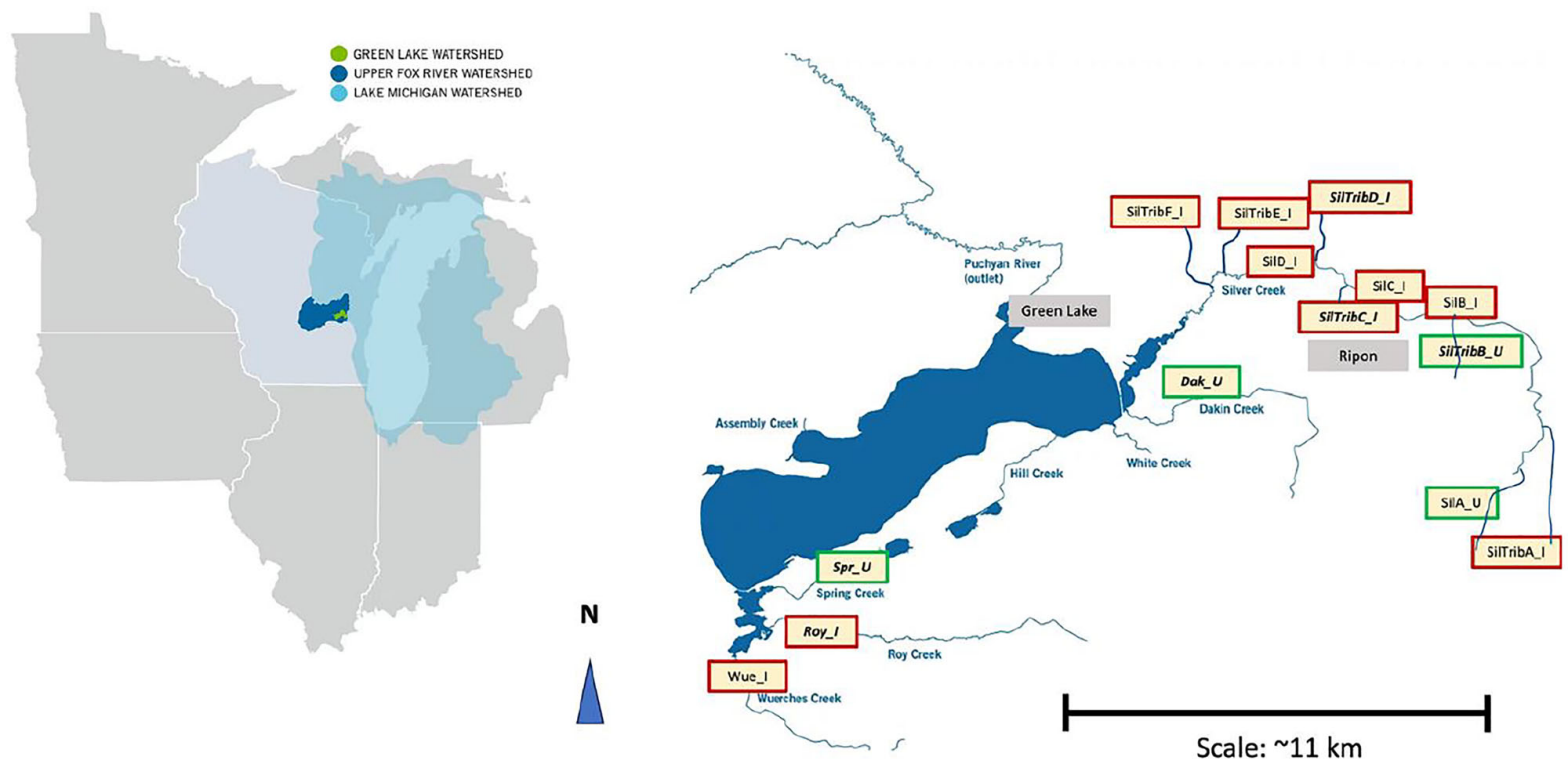
Map of Green Lake and its surrounding tributaries with the approximate locations of the 14 sample sites. Sites labeled “Sil” or “SilTrib” drain into the Silver Creek Estuary, and the County K Marsh area sample sites are Wue_I, Roy_I, and Spr_U on Wuerches, Roy, and Spring Creeks, respectively. Impaired sites are outlined in red, and unimpaired sites are outlined in green. Macroinvertebrates were sampled at six sites (bold italicized). The cities of Green Lake and Ripon are located near the gray boxes (original image by Green Lake Association, 2024).

Green Lake's average depth is 30.5 m (∼72 m at its deepest), and its retention time is 21 years (GLA, 2024). The lake's long retention time means that nutrient pollution problems may take a while to appear because water and pollutants enter the large lake slowly from its smaller tributaries, but once pollutants do enter, they also take a long time to flush out and resolve. The watershed, located in south‐central Wisconsin near the towns of Green Lake and Ripon, drains into the Upper Fox River Watershed and ultimately into Lake Michigan (Figure 1).

About 84% of all external total phosphorus (TP) loading into Green Lake comes from its tributaries (Fuller et al., 2022). Silver Creek is the largest tributary to Green Lake, which contributes about half of the external TP loading into the lake. Spring, Roy, and Wuerches Creeks encompass the County K Marsh area, which collectively contribute about 30% of the external TP loading into the lake (GLA, 2024). Of the 14 sample sites, 10 are listed as impaired, and four are unimpaired (Table 1).

**TABLE 1:** Sample site summary information, including the year listed as impaired for total phosphorus, the year of completed restoration or improvement project work, and the type of restoration or improvement project (if applicable). Data obtained from the WDNR Surface Water Data Viewer and the Green Lake Association, 2024.

Site	Name	Year listed as impaired	Restoration project completion	Type of project
SilA_U	Silver Creek at County KK	–	–	–
SilB_I	Silver Creek at Douglas Street (Hwy 44)	1998	–	–
SilC_I	Silver Creek DS Scott Creek Dam	1998	–	Streambank and dam updates
SilD_I	Silver Creek at Koro Road	1998	–	–
Dak_U	Dakin Creek West of FDL County Line	–	2021	Streambank and shoreline; brook trout
SilTribF_I	Unnamed tributary to Silver Creek at Hwy 23	2016	2020	Pedestrian bridge and streambank^a^
SilTribE_I	Unnamed tributary to Silver Creek at Murray Road	2016	–	–
SilTribD_I	Unnamed tributary to Silver Creek at Trail (County FF)	2016	2019	Pedestrian bridge and trail^a^
SilTribC_I	Unnamed tributary to Silver Creek at US Arcade Road	2018	–	–
SilTribB_U	Unnamed tributary to Silver Creek at Hwy 23	–	2018	Streambank and shoreline
SilTribA_I	Unnamed tributary to Silver Creek at County KK	2016	–	–
Wue_I	Wuerches Creek Upstream from County Road B	1998	–	–
Roy_I	Roy Creek downstream of County Hwy O	2002	2013	Streambank and shoreline; grade stabilization
Spr_U	Spring Creek upstream of County Hwy K	–	2013	Grade stabilization

^a^ These stream improvement projects are more focused on recreational use near or around the stream, not specifically on enhancing the stream hydrology or ecology.

Stream improvement projects have been implemented on seven of the 14 sample stream sites, located either at or upstream of the sample site. Stream improvement projects include streambank updates in response to cleaning up construction projects, dam maintenance, streambank and shoreline erosion control with riparian vegetation, grade stabilization, and pedestrian access such as trails or bridges. Each of the 14 sites was visited once in each season (summer and autumn) during both years (2021–2022). At every visit, three water quality samples were collected per site, and one macroinvertebrate sample was collected at six of the sites during the autumn season only. Site SilD_I was inaccessible in the 2022 seasons due to downed trees and high streamflow. Site SilTribF_I was inaccessible in the autumn 2021 season due to unsafe stream slope conditions and deep sticky muck in the streambed.

### 2.2 Data collection

#### 2.2.1 Biological data

Macroinvertebrate sampling followed the WDNR's guidelines and protocols for wadable streams and rivers (WDNR, 2017). Macroinvertebrate species can be used to analyze species assemblage composition and evaluate the status or trends of water quality and biological condition of streams and rivers (WDNR, 2017). Macroinvertebrate samples were taken at six sites over 2 years in the autumn (September through November) when water levels were near baseflow to detect cumulative summer and autumn water quality stressors (WDNR, 2017). Samples were collected in riffle habitats using a D‐frame 500‐ or 600‐µm mesh kick‐net and by facing upstream (WDNR, 2017). After streamside identification, the samples were preserved with ∼95% ethanol in the field and transported back to the lab for storage and any future identification needs (WDNR, 2017). For each site, the Citizen Monitoring for Biotic Index form was filled out (WDNR, 2017) to identify the abundances of species types and sensitivity groups to develop an index score for the site.

The Citizen Monitoring for Biotic Index approach, developed by the WDNR, focuses on the variety and types of macroinvertebrates and their tolerance to pollution rather than the number of animals found (WDNR, 2017). Macroinvertebrate species are divided into four groups, ranking from most sensitive to pollution (Group 1) to most tolerant to pollution (Group 4). Each type of animal that appears in the sample is circled on the sheet within each group. The index is developed by first adding the number of types of animals in each group, then adding one point to the group that has the most common animal (add zero to the other three groups). Each group is assigned a value (4 for Group 1, 3 for Group 2, 2 for Group 3, and 1 for Group 4), which places a heavier weight on more sensitive groups, creating a higher index score. The index score is then calculated by dividing the total value (the sum of the number of animals in each group multiplied by that group's value) by the total animals (the sum of all the different types of animals among all groups). The health of the stream based on the index score is rated “excellent” for scores 3.6 and above, “good” for scores rated 2.6–3.5, “fair” for scores rated 2.1–2.3, and “poor” for scores between 1 and 2.

#### 2.2.2 Field environmental data

At each sample site, water temperature, transparency, and DO were recorded, and water samples (three samples per site) were collected from the middle, left bank, and right bank within the stream in a 2‐L plastic container that was brought back to the laboratory for chemistry analysis (pH, total solids [TS], nitrate, total Kjeldahl nitrogen [TKN], and TP) (WDNR, 2015). The water samples were transported and stored at 4°C until analysis. DO (mg/L) and water temperature (°C) were recorded using a handheld DO meter with a Teflon membrane‐covered polarographic sensor and a 15‐m‐long cable (Yellow Springs Inst. Co. 550A‐12). The YSI was calibrated for DO in the field prior to each sampling event, and the sensor has a precision of about +0.2 mg/L. In addition to monitoring chemical parameters within each stream, the surrounding stream habitat at each sample site was evaluated using the WDNR Wadable Stream Qualitative Fish Habitat Rating for Streams <10 m wide (WDNR, 2015). Ratings on riparian buffer width (m), bank erosion, pool area, width:depth ratio, riffle:bend ratio, fine sediments, and cover for fish were recorded and averaged for a final score ranging from 0 (poor), 33 (fair), 67 (good), to 90 (excellent).

#### 2.2.3 Qualitative environmental and site data

Stream quality classifications are determined by whether they have had completed stream improvement work, whether the stream is impaired, and the site's habitat quality. Of the 14 total sample sites, six have had completed restoration or stream improvement work (sites Dak_U, SilTribF_I, SilTribD_I, SilTribB_U, Roy_I, and Spr_U) and eight have not (sites SilA_U, SilB_I, SilC_I, SilD_I, SilTribE_I, SilTribC_I, SilTribA_I, and Wue_I) (Table 1). The WDNR Surface Water Data Viewer maps impaired water bodies throughout the state and can identify water bodies such as streams and rivers that are impaired. Tributaries that are impaired in the Green Lake Watershed are listed due to exceeding phosphorus thresholds. Sites will be classified as impaired (Sites SilB_I, SilC_I, SilD_I, SilTribF_I, SilTribE_I, SilTribD_I, SilTribC_I, SilTribA_I, Wue_I, and Roy_I) or unimpaired (SilA_U, Dak_U, SilTribB_U, and Spr_U) (Table 1 and Figure 1). The Wisconsin State threshold for TP in surface water streams is 0.075 mg/L (WDNR, 2015). Sites that exceeded this threshold at the time of sampling will be noted for reference. Habitat classification is based on rating scores (Section 2.2.2).

### 2.3 Water quality analysis

All water samples were analyzed within 48 h after collection. Chemistry analysis included pH, TS, TP, TKN, and nitrate (WDNR, 2015). Prior to acidification with 2 mL of sulfuric acid per liter of sample, the pH of each sample was determined immediately upon returning from the field using a standard electric probe (SevenExcellence pH Meter S400). Each sample was then divided into two bottles: one for the raw sample and one that was acidified with 2 mL of sulfuric acid per liter of sample to prepare for TP and TKN analysis. The TS in raw water samples were determined following the standard methods of the American Public Health Association (APHA, 1995).

The acidified samples were digested following the standard methods of the Environmental Protection Agency (Methods 351.2 and 365.1) and analyzed for TP and TKN calorimetrically using a SEAL AQ2 Discrete Analyzer (SEAL Analytical, 2015a, 2015b; US EPA, 1993a, 1993b). Duplicates of at least every four samples were included to test for accuracy in analysis, with a +10% acceptable deviation from the original (SEAL Analytical, 2015a, 2015b).

The remaining raw samples were filtered to prepare for nitrate analysis with a single reagent made from HCl, *N*‐(1‐naphtyl)ethylenediamine dihydrochloride (NEDD), vanadium (III) chloride (VCl_3_), and sulfanilamide (Doane & Horwáth, 2003) modified for microplate analysis. Appropriate 275 µL aliquots of water samples and reagent volumes were pipetted into cuvettes and incubated for 14–16 h for maximum development (Doane & Horwáth, 2003), then analyzed for absorbance using a microplate reader at 540 nm within 48 h of incubation. The absorbance was calibrated to determine the nitrate concentration (mg/L) of each sample, including duplicates for every four samples. For the purposes of this study, total nitrogen (TN) consists of TKN and nitrate, and neglects nitrite because concentrations of nitrite in stream plants and water are low relative to nitrate concentration, as nitrite is easily oxidized to nitrate (Daims et al., 2016). Nitrate and TKN were obtained to quantify TN in water samples, and therefore, only TN was included in statistical analyses.

### 2.4 Data analysis

Two main categories of data were analyzed in this study. Stream quality classifications are categorical independent variables (impairment [yes/no], habitat category [good, fair, and poor], and stream improvement [yes/no]), and water quality parameters (TP, TN, DO, pH, habitat rating, and TS), and invertebrate indices are dependent numerical variables. “Habitat category” and “habitat rating” are related but two distinct variables in this analysis. Data collected from 2021 to 2022 were analyzed for water quality parameter reflections of stream classification, invertebrate response to water quality parameters, and invertebrate reflections of stream classification.

In practice, water quality parameters generally determine stream quality classification (e.g., impairment is determined by continuous exceedance of phosphorus concentrations), but the Kruskal–Wallis rank sum test uses independent categorical variables and dependent numerical variables. For the purposes of this analysis, which assesses how these variables reflect one another, stream quality classifications are considered independent variables and water quality parameters are considered dependent variables. Kruskal–Wallis rank sum tests were used to determine significant differences (*p*‐value < 0.05) in stream quality classifications based on water quality parameters, as well as significant differences between invertebrate indices and stream quality classifications. These tests were also used to check for any differences between water quality parameters and the two sampling years (2021–2022) and between sampling seasons (summer and autumn). Analysis of similarities (ANOSIM) was used to determine significant dissimilarities between invertebrate indices and stream classification status, as well as water quality parameters. The data were not normally distributed, so ANOSIM was used as a nonparametric alternative to permutational multivariate analysis of variance. Using the Vegan package in R, an ordination process was used to scale down the dimensions of the data and parameters. Vegan is a package for community ecologists that supports all basic ordination methods and functions for fitting environmental variables to ordination graphics (Oksanen, 2015; 2022). Nonmetric multidimensional scaling (NMDS) techniques were used to order stream sites based on macroinvertebrate indices. NMDS is used to establish ordination objects between biotic indices and sample sites, which can then be compared to water quality parameters (Oksanen, 2022). Examining the correlating water quality parameters with NMDS axis scores allowed us to observe relations between these variables to invertebrate indices.

## 3 RESULTS

### 3.1 Summary statistics of water quality data

In the summer sampling seasons at the time of sampling, 11 sites exceeded the Wisconsin TP criterion (>0.075 mg/L) in 2021, and eight sites exceeded the criterion in 2022 (Table 2) (WDNR, 2015).

**TABLE 2:** Water quality parameters (mean ± standard deviation) at each sample site during the summer season (August).

Site^a^	Year	TP (mg/L)^b^	TN (mg/L)	DO (mg/L)^c^	pH^c^	TS (mg/L)	Habitat rating^c^	Transparency (cm)^c^
SilB_I	2021 2022	0.21* (0.02) 0.07 (0.06)	3.03 (0.08) 6.89 (0.13)	2.02 –	– –	325.7 (10.3) 378.6 (42.8)	67 75	– 113
SilC_I	2021 2022	0.22* (0.01) 0.11* (0.03)	3.99 (0.76) 9.93 (0.69)	6.52 –	9.00 –	350.5 (4.4) 407.2 (14.3)	62 72	– 120
SilD_I	2021 2022	0.17* (0.01) –	3.73 (0.53) –	6.70 –	8.10 –	83,028.6 (9.9) –	70 –	– –
SilTribF_I	2021 2022	0.16* (0.08) 0.20* (0.04)	18.85 (0.12) 11.41 (0.83)	6.85 –	– –	83,059.0 (49.0) 592.9 (35.9)	60 67	– 21
SilTribE_I	2021 2022	0.06 (0.03) 0.10* (0.02)	4.41 (0.51) 1.58 (0.70)	6.25 5.50	– 8.50	82,901.9 (9.1) 378.6 (14.3)	60 63	– 120
SilTribD_I	2021 2022	0.27* (0.02) 0.55* (0.13)	3.67 (0.71) 2.80 (0.12)	1.73 1.50	7.50 7.00	82,921.7 (7.2) 438.0 (15.6)	75 82	– 71
SilTribC_I	2021 2022	0.11* (0.03) 0.21* (0.08)	2.97 (0.11) 1.30 (0.28)	7.40 7.00	8.20 8.10	83,018.1 (21.6) 467.0 (28.6)	50 53	– 68
SilTribA_I	2021 2022	0.17* (0.00) 0.21* (0.12)	3.24 (0.23) 5.36 (0.32)	4.56 –	– –	378.6 (82.1) 474.3 (38.4)	40 52	– 111
Wue_I	2021 2022	0.11* (0.02) 0.04 (0.01)	11.60 (0.34) 19.19 (0.07)	7.03 9.50	6.00 7.00	523.8 (4.4) 471.4 (49.5)	68 63	– 120
Roy_I	2021 2022	0.12* (0.02) 0.17* (0.03)	9.62 (0.27) 16.00 (0.16)	9.01 9.50	8.30 7.00	487.6 (4.3) 438.0 (33.0)	82 67	– 19
SilA_U	2021 2022	0.18* (0.04) 0.09* (0.01)	4.11 (0.43) 2.34 (0.49)	4.66 –	8.00 –	485.7 (75.6) 392.9 (27.4)	53 80	– 84
Dak_U	2021 2022	0.11* (0.01) 0.07 (0.05)	13.51 (0.43) 27.00 (0.13)	10.41 11.00	8.10 7.00	476.2 (10.8) 486.0 (23.4)	65 70	– 102
SilTribB_U	2021 2022	0.05 (0.03) 0.01 (0.01)	15.40 (0.29) 17.00 (0.15)	7.36 7.20	7.30 7.00	438.1 (143.8) 552.0 (14.3)	48 60	– 78
Spr_U	2021 2022	0.01 (0.00) 0.04 (0.03)	5.52 (0.40) 6.30 (0.14)	7.15 7.00	7.90 7.80	344.8 (49.2) 267.0 (12.8)	85 95	– 118

Abbreviations: TN, total nitrogen; TS, total solids.

^a^ Impaired sites end with “_I” and unimpaired sites end with “_U.”

^b^ Total phosphorus (TP) concentrations marked with an asterisk (*) are above the Wisconsin State threshold (0.075 mg/L) at the time of sampling.

^c^ Dissolved oxygen (DO), pH, habitat rating, and transparency were only measured once per site in each year.

Macroinvertebrates were sampled at sites Dak_U, SilTribD_I, SilTribC_I, SilTribB_U, Roy_I, and Spr_U in both years during the autumn season (detailed taxa information found in Table S1). Index scores ranged from 1.67 to 3.50 across all sampled sites. At the time of sampling, seven sites exceeded the phosphorus criterion (0.075 mg/L) in the autumn 2021 season, and eight sites exceeded the criterion in the autumn 2022 season (Table 3).

**TABLE 3:** Water quality parameters (mean ± standard deviation) and macroinvertebrate index scores at each sample site during the autumn season (October–November).

Site^a^	Year	TP (mg/L)^b^	TN (mg/L)	DO (mg/L)^c^	pH^c^	TS (mg/L)	Habitat rating^c^	Trans. (cm)^c^	Biotic index
SilB_I	2021 2022	0.07 (0.03) 0.03 (0.05)	9.87 (0.49) 10.00 (0.77)	– –	8.00 –	552.4 (115.5) 478.6 (85.3)	48 45	40 107.5	– –
SilC_I	2021 2022	0.04 (0.02) 0.03 (0.04)	9.06 (0.26) 7.79 (0.30)	– –	8.30 –	327.5 (249.6) 400.0 (0.0)	47 52	78 59	– –
SilD_I	2021 2022	0.12* (0.02) –	8.98 (0.40) –	7.00 –	8.10 –	635.7 (14.3) –	53 –	33 –	– –
SilTribF_I	2021 2022	– 0.12* (0.06)	– 6.90 (0.63)	– –	– –	– 435.7 (42.9)	– 65	– 43	– –
SilTribE_I	2021 2022	0.07 (0.01) 0.02 (0.05)	4.48 (0.42) 1.37 (0.07)	– –	7.80 –	409.5 (16.5) 350.0 (14.3)	32 60	13 120	– –
**SilTribD_I**	2021 2022	0.12* (0.03) 0.24* (0.09)	2.83 (0.16) 1.98 (0.11)	1.50 3.00	7.50 7.00	428.6 (0.0) 442.9 (16.5)	60 62	55 111	1.83 2.67
**SilTribC_I**	2021 2022	0.16* (0.15) 0.09* (0.04)	3.20 (0.47) 2.57 (0.08)	– –	8.20 –	533.3 (59.5) 514.3 (57.2)	33 85	65 120	3.13 3.00
SilTribA_I	2021 2022	0.20* (0.06) 0.12* (0.07)	4.43 (0.41) 4.67 (0.10)	– –	7.80 –	564.3 (112.8) 521.5 (27.4)	30 33	46 19	– –
Wue_I	2021 2022	0.06 (0.01) 0.15* (0.10)	13.68 (0.22) 13.42 (0.26)	7.50 –	7.80 –	504.8 (33.0) 592.9 (14.3)	55 77	95 120	– –
**Roy_I**	2021 2022	0.09* (0.01) 0.09* (0.12)	17.58 (0.41) 13.13 (0.36)	10.00 12.50	8.30 7.00	504.8 (43.7) 528.6 (49.5)	45 50	76 106.5	3.00 3.40
SilA_U	2021 2022	0.27* (0.29) 0.61* (0.13)	4.03 (1.44) 6.74 (2.56)	8.00 –	7.60 –	485.7 (0.0) 807.3 (42.9)	50 80	40 21	– –
**Dak_U**	2021 2022	0.09* (0.08) 0.04 (0.03)	12.41 (0.47) 18.81 (0.75)	– –	8.10 –	533.3 (108.2) 507.1 (42.9)	55 62	47 117	3.50 3.20
**SilTribB_U**	2021 2022	0.07 (0.02) 0.05 (0.04)	18.73 (0.10) 10.14 (0.11)	– –	7.30 –	533.3 (33.0) 557.1 (28.6)	25 35	92 120	1.67 3.17
**Spr_U**	2021 2022	0.02 (0.00) 0.13* (0.16)	4.85 (0.18) 4.44 (0.30)	– –	7.90 –	414.3 (82.5) 335.7 (148.2)	58 63	102 109	3.00 3.14

Abbreviations: TN, total nitrogen; TS, total solids.

^a^ Impaired sites end with “_I” and unimpaired sites end with “_U.” Macroinvertebrates were only sampled at sites in bold.

^b^ Total phosphorus (TP) concentrations marked with an asterisk (*) are above the Wisconsin State threshold (0.075 mg/L) at the time of sampling.

^c^ Dissolved oxygen (DO), pH, habitat rating, and transparency (Trans.) were only measured once per site in each year.

### 3.2 Stream water quality associations with overall stream quality

The null hypothesis that water quality parameters did not reflect overall stream quality is rejected for some water quality parameters and impairment status, habitat quality, and whether stream improvement projects have been implemented at or upstream from the site (Table 4). The Kruskal–Wallis results with significant water quality parameter associations to stream classifications are summarized in Table 4.

**TABLE 4:** The Kruskal–Wallis rank sum test results of how significant (*p*‐value < 0.05) water quality parameters differ among stream classification variables.

Stream quality classification	Water quality parameter(s)	*χ* ^2^	*df*	*p*‐value
Impairment status	Total phosphorus (mg/L)	16.017	1	<0.0001
	Total nitrogen (mg/L)	10.315	1	0.0013
	Dissolved oxygen (mg/L)	4.2412	1	0.039
Habitat quality	Total phosphorus (mg/L) pH Total solids (mg/L)	14.018 24.535 14.662	3 3 3	0.0029 <0.0001 0.0021
	Transparency (cm)	13.063	3	0.0045
	Habitat rating	146.6	3	<0.0001
Stream improvement	Total nitrogen (mg/L) Stream flow (cfs) Habitat rating	26.888 4.7033 6.5865	1 1 1	<0.0001 0.030 0.010
Year^a^	pH Total solids (mg/L) Habitat rating	20.253 9.2142 21.739	1 1 1	<0.001 0.0024 <0.001
Season^a^	Total phosphorus (mg/L) pH Habitat rating	7.1265 5.9163 42.064	1 1 1	0.0076 0.015 <0.001

*Note*: “*χ* ^2^” refers to the chi‐squared value, and “*df*” refers to degrees of freedom.

^a^ The sampling year and season are not stream classification variables but are included in the analysis to compare variables that differed between 2021 and 2022 (year) or summer and autumn (season).

There were a few differences between water quality parameters and sampling year or season when comparing concentrations at each site. TS differed between sampling years (generally 2%–24% higher concentrations in 2021 than in 2022) but were consistent between the summer and autumn seasons in both years. TP differed between summer and autumn, with 17%–80% lower concentrations in autumn, but was consistent between the summers of 2021 and 2022, and between the autumns of 2021 and 2022. Habitat rating and pH differed between both summer and autumn seasons as well as between both sampling years. Habitat ratings were 4%–50% higher in 2022 than in 2021, and 3%–57% higher in the summer seasons than in the autumn seasons. pH was generally 7%–12% higher in 2021 than in 2022, and 0.01%–26% higher in the autumn seasons than in the summer seasons.

### 3.3 Water quality associations with macroinvertebrate indices

**FIGURE 2:**
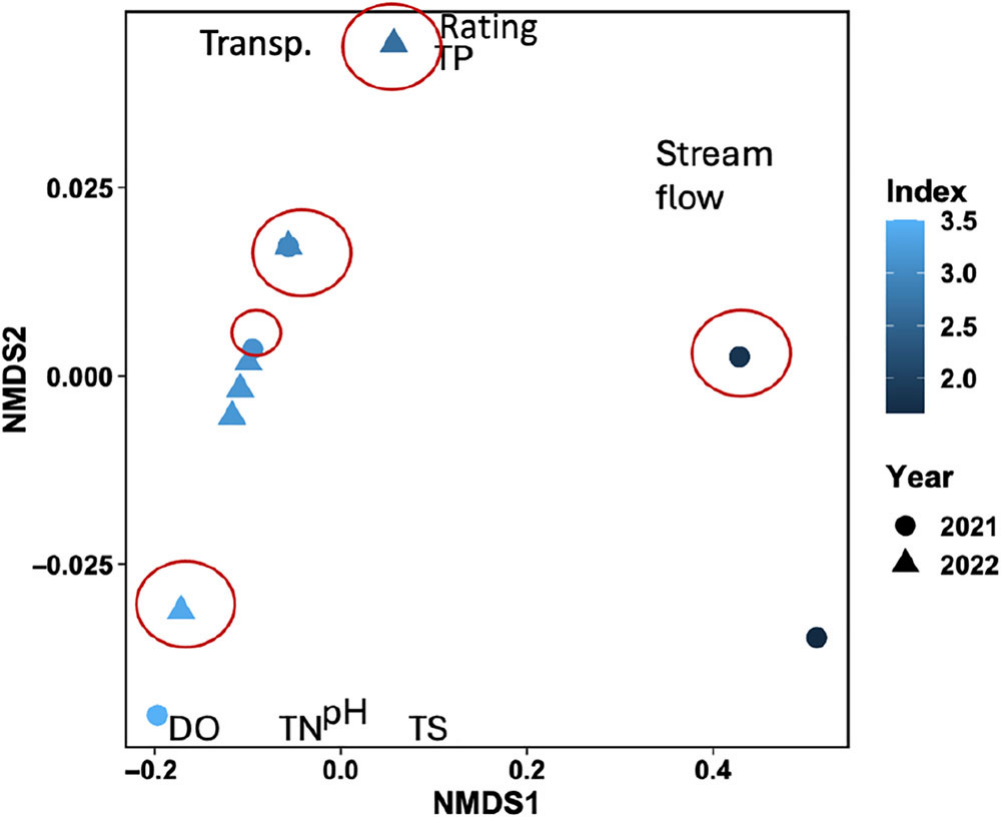
Nonmetric multidimensional scaling (NMDS) plot of invertebrate indices at each sample site in 2021 and 2022, and the ordination of water quality parameters, based on their NMDS coordinates (see Table S2). NMDS axes represent a metric of relatedness between water quality parameters and invertebrate indices based on their proximity to one another. Darker shades of blue indicate a lower index score, circles and triangles represent the sampling years of 2021 and 2022, respectively, and sites circled in red are listed as impaired. DO, dissolved oxygen; TN, total nitrogen; TP, total phosphorus; TS, total solids.

Invertebrate indices were also significantly different between sampling years (ANOSIM *R* = 0.1722, *p* = 0.001) and between sample sites (*R* = 0.4102, *p* < 0.0001). NMDS analysis was used for the autumn 2021 and 2022 sampling data to determine whether macroinvertebrate indices associate with water quality parameters. The Kruskal–Wallis rank sum test unveiled that macroinvertebrate indices varied significantly between sampling years (*χ* ^2 ^= 5.9014, *df* = 1, *p*‐value = 0.015). Figure 2 summarizes the NMDS visualization of the relatedness between the macroinvertebrate index and water quality parameters at each of the six sample sites.

NMDS axes represent a metric of relatedness between parameters based on their proximities to one another within the plot. Parameters that are closer together on the plot are more related. Macroinvertebrates tended to associate more positively with higher DO concentrations and lower TP concentrations. ANOSIM analysis unveiled that the invertebrate index was significantly different from TP (*R* = 0.2375, *p*‐value = 0.014), TN (*R* = 0.8962, *p*‐value = 0.013), TS (*R* = 0.1135, *p*‐value = 0.047), habitat rating (*R* = 0.6815, *p*‐value < 0.0001), and transparency (*R* = 0.8538, *p*‐value < 0.0001). When TP concentrations were high, invertebrate indices tended to be lower, though the opposite was true for TN.

### 3.4 Macroinvertebrate index reflections of stream quality

**FIGURE 3:**
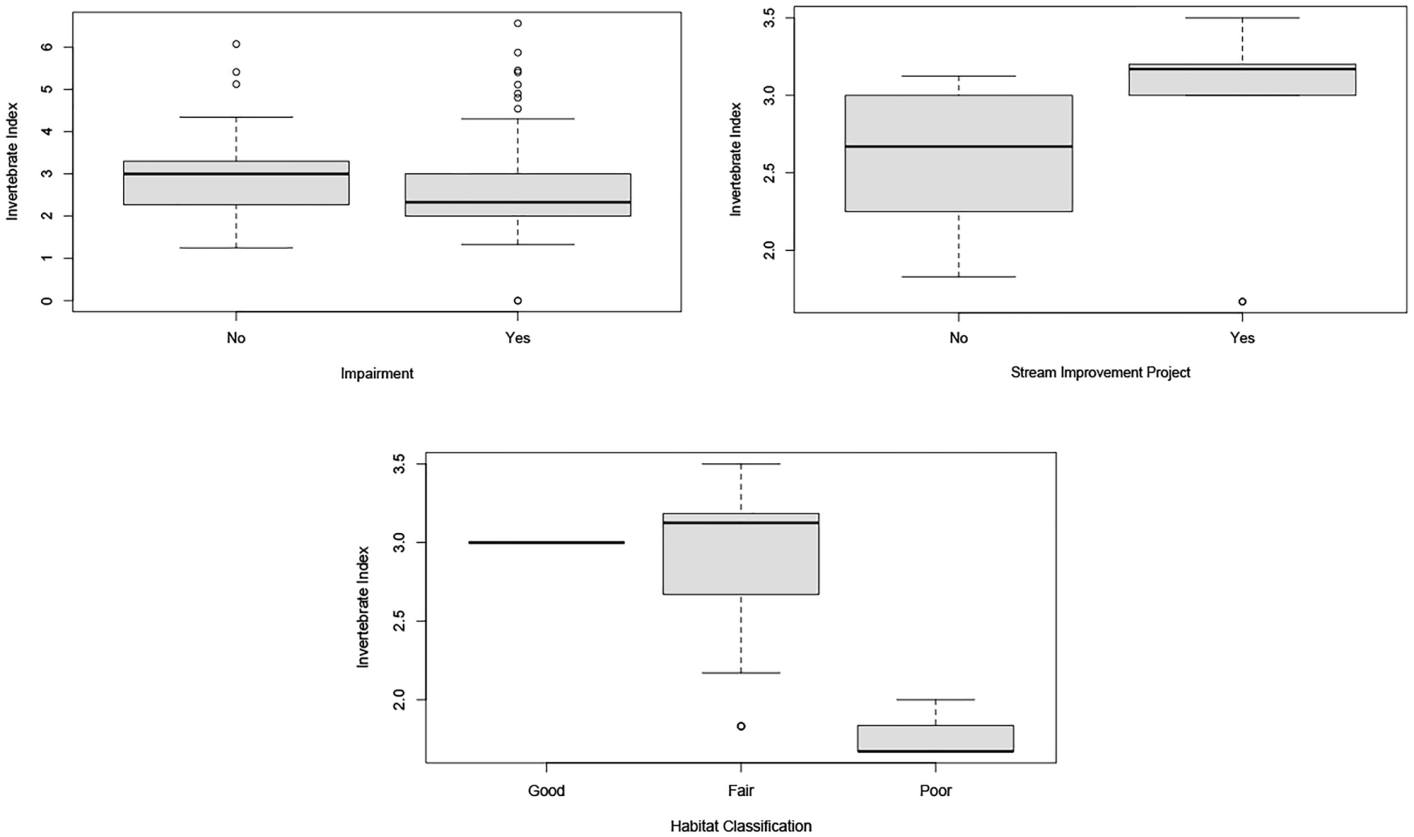
Boxplots of the invertebrate index in streams that are impaired and unimpaired (top left), streams with and without improvement projects (top right), and three different habitat classifications (good, fair, and poor) based on the habitat rating scores (bottom).

To determine whether macroinvertebrate indices reflect stream quality classifications at each of the six sample sites, the Kruskal–Wallis rank sum test was used with the 2021–2022 sampling data. Invertebrate index scores varied significantly between impaired and unimpaired streams (Kruskal–Wallis *χ* ^2^ = 7.8388, *df* = 1, *p*‐value = 0.0051; ANOSIM *R* = 0.0764, *p*‐value = 0.017), as well as between streams with restoration or improvement projects and streams without such projects (Kruskal–Wallis *χ* ^2^ = 11.834, *df* = 1, *p*‐value = 0.00058). In general, invertebrate indices were higher in unimpaired sites and in sites that had implemented improvement projects (Figure 3).

Biotic index scores also varied significantly with habitat classification (Kruskal–Wallis *χ* ^2^ = 15.64, *df* = 3, *p*‐value = 0.0013; ANOSIM *R* = 0.2939, *p*‐value = 0.020), though habitat data may be limited due to fewer streams overall falling within the “good” and “poor” classifications (Figure 3).

## 4 DISCUSSION

The goal of this study was to first assess overall water quality across a Wisconsin agricultural watershed, then provide some foundational analysis that investigates how indices may reflect stream quality classification. This study demonstrated that water quality parameters can reflect stream quality, including stream restoration. Impaired tributaries in Wisconsin are generally listed due to high TP concentrations, high TS loading, or both, but are listed based on TP concentrations in the Green Lake Watershed. Impairment status was reflected through TP and TN concentrations and DO at all sample sites, though not TS or transparency. Transparency, a measurement of water clarity, is affected by TS in the water column, which can also transport particulate phosphorus from erosion and soil loss in runoff. Dissolved phosphorus entering tributaries from crop fertilizer applications also has the potential to limit transparency by temporarily increasing macrophyte abundance or promoting algal blooms (Hamid et al., 2020). Since TS was not a significant factor in reflecting impairment status at the time of sampling, much of the TP concentrations at the sample sites may have been in the dissolved form rather than the particulate form. The adsorption, assimilation, and release of phosphorus in aquatic ecosystems are determined by environmental conditions such as pH (Zhao et al., 2022). Agricultural watersheds tend to have higher acidity in receiving tributaries (Hamid et al., 2020). Phosphorus release from sediment increases with rising pH, and the pH levels in both seasons from 2021 to 2022 were generally alkaline or neutral, ranging from 6 to 9 in the summer seasons and 7 to 8.3 in the autumn seasons. The sample sites with generally the highest pH in both seasons were in closer proximity to downtown Ripon relative to other sites, and urban stormwater runoff has the potential to increase pH in receiving waters (Asabere et al., 2018). Calcium and magnesium, common minerals found in the watershed due to sandstone and dolomite bedrock (City of Madison, 2024), can also contribute to raising pH in streams and rivers (Kubisch & Korff, 2023). Further studies are recommended to determine any statistical linkages between stream pH and phosphorus release in the watershed.

Macroinvertebrate indices reflected some water quality parameters in autumn. Higher TP concentrations (as well as lower DO measurements) indicated lower indices, and higher TN concentrations indicated higher indices. We found a similar relationship with DO but contrasting findings with TP when assessing a larger dataset for the watershed over a longer time period (2002–2022). Wisconsin's Surface Water Integrated Monitoring System (SWIMS) database showed that invertebrate indices were predicted to increase with increasing seasonal averages of DO as well as increasing seasonal averages of TP measurements (see Table S3 and Figures S1–S3). Though incorporating relationships from larger databases like SWIMS is useful to help assess long‐term trends and provide additional data, not all water quality parameters or invertebrate indices were sampled at all sites in all years, or all at the same time within seasons (Figure S4). Seasonal averages of indices and water quality parameters in the larger database were used to help mitigate these challenges in comparing relationships to data collected in this study. Both nitrogen and phosphorus are commonly identified as predictors of macroinvertebrate integrity (Heatherly et al., 2007; Stone et al., 2005; Wang et al., 2007), though not always in consistent ways. In Wang et al.’s (2007) study on wadeable streams in Wisconsin, macroinvertebrate indices increased with lower phosphorus and nitrogen concentrations, but Stone et al.’s (2005) study showed an increase in indices with increasing orthophosphate concentration in Illinois streams. Another study conducted in Illinois streams (Heatherly et al., 2007) showed a decrease in macroinvertebrate communities with increasing nutrient (phosphorus and nitrogen) pollution but concluded that habitat quality factors also need to be considered in index fluctuations. Macroinvertebrate assemblages have also been used to establish the Wisconsin TP threshold (Wang et al., 2007). In agriculturally dominated streams like Green Lake's tributaries, nitrogen concentrations can be sufficiently high to preclude nitrogen limitation of algae, which suggests that phosphorus may be the critical nutrient affecting macroinvertebrate assemblages within the sample sites (Heatherly et al., 2007).

Macroinvertebrate indices can also reflect stream quality, including stream restoration. Measured in autumn, biotic index scores were on average higher in streams that had improvement projects like riparian restoration than in streams without projects in this study. Indices were also significantly different with habitat classification as index scores were substantially lower in sites rated as “poor” habitat quality, though they were also on average higher in sites rated as “fair” than in sites rated as “good.” The analysis revealed a statistically significant difference in overall habitat classification. Macroinvertebrate communities are known to reflect ambient environmental conditions (Stone et al., 2005), as higher densities of sensitive species are typically associated with higher‐quality stream conditions (Barbour et al., 1999; Hilsenhoff, 1987, 1988; Lenat, 1993; Stone et al., 2005). Higher quality sites, such as those within forested land use areas, are typically going to promote more biodiversity than lower quality sites like those in urban and agricultural watersheds. In‐stream habitat quality is cited to be the most important limiting factor for biotic integrity in aquatic ecosystems (Stone et al., 2005), and macroinvertebrate assemblages are strongly linked to physical parameters in and around streams (Wang et al., 2007). Another study in Illinois concludes that habitat degradation is generally evident in streams with higher nutrient concentrations, and habitat was not the primary factor for biotic integrity for some agricultural watersheds (Heatherly et al., 2007). There was a clear distinction between “poor” classified sites (mostly urban land use areas) and other classifications with exceptionally low indices, but compounding factors with nutrients made the distinction between “fair” agricultural sites and “good” forested sites less distinct (Heatherly et al., 2007). Expanding the sample size in future studies could help clarify distinctions in biotic indices between sites classified as “good” and “fair.”

This study compared indices at sites with and without restoration projects and no changes before and after restoration project completion. Analysis of macroinvertebrate indices before and after restoration project completion would reveal insights on the performance and effectiveness of said project. Further research on before and after comparisons would be useful to evaluate restoration overall, but it is important to first consider a baseline understanding of how sites with and without restoration projects affect macroinvertebrate indices, and how that ties into overall stream habitat quality. A limitation is that sites that have not had any stream improvement projects may not have poor habitat quality or may already have a heavy riparian vegetation influence, and sites with such projects likely have higher habitat quality in general. Still, there is potential in using forested riparian buffers to protect and improve stream health, as even small amounts of riparian forest cover can be associated with increased biodiversity and in‐stream habitat quality (Stone et al., 2005). Some macroinvertebrate species, such as caddisflies, mayflies, and dobsonflies that are grazers and shredders, consume organic material within streams that are provided by the surrounding landscape (see Table S1). Crops using pesticides might have chemicals stored in their organic material that ultimately can get ingested by these invertebrates and lower their diversity within streams (Stone et al., 2005). Although macroinvertebrates were not measured in the summer, Green Lake's agricultural land use is mostly cropping agriculture, which can contribute to overall lower index scores in the autumn if pesticides or fertilizers are used even in areas with higher riparian vegetation or higher habitat quality. Future research that analyzes these functional traits and how they relate to stream restoration in agricultural watersheds would further enhance the understanding of restoration effectiveness.

Additional studies that analyze seasonal variations in nutrient concentrations would be useful to further draw conclusions about the effect of riparian vegetation and seasonal variation on stream water quality throughout the year. Nutrient concentrations fluctuated significantly between seasons and baseflow conditions. The effects of stream restoration on water quality are subject to seasonal variability (Chen et al., 2021), as nutrient concentrations tend to be higher in the summer. Increased nutrient concentrations in streams lead to increases in microbial biomass, which consumes more oxygen (Hamid et al., 2020). When TP concentrations in this study are high, DO measurements are low likely due to the increase in algae respiration. Generally, when sites exhibited high TP concentrations, they had low TN concentrations, and vice versa. Sources of TN in Green Lake's watershed come from crop fertilizer and groundwater interactions through springs (Prellwitz et al., 2022), though additional studies would be useful to further assess groundwater contributions. When one nutrient is limiting, the other nutrient tends to thrive, which could explain the inverse relationship between TP and TN. Generally, TP loads increase as TS loads increase (Fuller et al., 2022), and TN concentrations were lower with higher TP concentrations. Since TP and TS correlate with one another, when TN concentrations are lower, TS concentrations are likely to be higher. Higher streamflow rates also help circulate water to clear out nutrients and other pollutants (Hamid et al., 2020), so TN concentrations are generally observed to be higher in sites with lower streamflow.

This research highlights gaps in the frequency and types of water quality monitoring and can guide future efforts to assess stream restoration outcomes and their relationship to biological integrity in agriculturally influenced lake watersheds. Identified gaps in using large datasets like SWIMS include the types of parameters that get sampled at different frequencies and temporal scales. The results from this study provide a foundational starting point for investigating stream improvement projects and their impact on biological species and water quality parameters. This research underscores the critical need for comprehensive and consistent water quality monitoring, offering a roadmap for future assessments to prioritize stream improvement initiatives, enhance public data synthesis, and better understand water quality's impact on biological integrity within agriculturally dominated watersheds.

## AUTHOR CONTRIBUTIONS

**Laura M. Bates**: Conceptualization; data curation; formal analysis; funding acquisition; investigation; methodology; project administration; software; visualization; writing—original draft; writing—review and editing. **Anita M. Thompson**: Conceptualization; funding acquisition; methodology; project administration; resources; supervision; validation; writing—review and editing. **Laxmi R. Prasad**: Investigation; methodology; resources; supervision; validation; writing—review and editing

## CONFLICT OF INTEREST STATEMENT

The authors declare no conflicts of interest.

## Supporting information



Supplemental materials are available and include: (i) a table of the list of taxa present at each sample site, (ii) a table of the NMDS analysis results coordinates, (iii) summarized analysis from 2002 to 2022 of a larger dataset from Wisconsin's Surface Water Integrated Monitoring System database to support findings in the manuscript discussion, (iv) a table of linear regression results comparing macroinvertebrate indices with some water quality parameters from this larger dataset, and (v) four figures that visualize this supplemental analysis.

## Data Availability

Data are available from the authors upon reasonable request.

## References

[jeq270122-bib-0001] APHA . (1995). Standard methods for the examination of water and wastewater (19th ed.). American Public Health Association.

[jeq270122-bib-0002] Asabere, S. , Zeppenfeld, T. , Nketia, K. , & Sauer, D. (2018). Urbanization leads to increases in pH, carbonate, and soil organic matter stocks of arable soils of Kumasi, Ghana (West Africa). Frontiers in Environmental Science, 6, Article 119. 10.3389/fenvs.2018.00119

[jeq270122-bib-0003] Barbour, M. T. , Gerritsen, J. , Snyder, B. D. , & Stribling, J. B. (1999). Rapid bioassessment protocols for use in wadeable streams and rivers: Periphyton, benthic macroinvertebrates, and fish (EPA 841‐B‐99‐002) (2nd ed.). USEPA.

[jeq270122-bib-0005] Cahill, M. , Emanuel, R. , Gilbertson, T. , Harlan, C. , Hottenroth, D. , Petersen, C. , Richardson, D. , Shaloum, G. , & Stoughton, C. (2013). Field guide: Managing rain gardens, swales and stormwater planters. Oregon State University Stormwater Solutions. https://extension.oregonstate.edu/stormwater‐green‐infrastructure?reference=sites/default/files/fieldguide.pdf

[jeq270122-bib-0006] Carpenter, S. , Booth, E. , & Kucharik, C. (2018). Extreme precipitation and phosphorus loads from two agricultural watersheds. Limnology and Oceanography, 63(3), 1221–1233. 10.1002/lno.10767

[jeq270122-bib-0007] Carpenter, S. R. , Caraco, N. F. , Correll, D. L. , Howarth, R. W. , Sharpley, A. N. , & Smith, V. H. (1998). Nonpoint pollution of surface waters with phosphorus and nitrogen. Ecological Applications, 8(3), 559–568. 10.2307/2641247

[jeq270122-bib-0009] Chen, Y. , Viadero, R. C. , Wei, X. , Fortney, R. , Hedrick, L. B. , Welsh, S. A. , Anderson, J. T. , & Lin, L.‐S. (2009). Effects of highway construction on stream water quality and macroinvertebrate condition in a mid‐Atlantic Highlands watershed, USA. Journal of Environmental Quality, 38(4), 1672–1682. 10.2134/jeq2008.0423 19549944

[jeq270122-bib-0058] Chen, Y. , Wang, Y. , Chia, B. , & Wang, D. (2021). Upstream‐downstream water quality comparisons of restored channelized streams. Ecological Engineering, 170, 106367. 10.1016/j.ecoleng.2021.106367

[jeq270122-bib-0010] City of Madison . (2024). Water utility—Frequently asked questions . https://www.cityofmadison.com/water/water‐quality/faq#:~:text=Madison%20tap%20water%20is%20very,not%20harmful%20to%20human%20health

[jeq270122-bib-0012] Craig, R. , & Roberts, A. (2015). When will governments regulate nonpoint source pollution? A comparative perspective. Boston College Environmental Affairs Law Review, 42(I), 1–64.

[jeq270122-bib-0013] Daims, H. , Lücker, S. , & Wagner, M. (2016). A new perspective on microbes formerly known as nitrite‐oxidizing bacteria. Trends in Microbiology, 24(9), 699–712. 10.1016/j.tim.2016.05.004 27283264 PMC6884419

[jeq270122-bib-0014] Doane, T. A. , & Horwáth, W. R. (2003). Determination of nitrate with a single reagent. Analytical Letters, 36(12), 2713–2722. 10.1081/AL-120024647

[jeq270122-bib-0015] Fuller, S. , Boswell, E. , Thompson, A. , & Robertson, D. (2022). Water‐quality improvement of an agricultural watershed marsh after macrophyte establishment and point‐source reduction. Wetlands, 42, Article 129. 10.1007/s13157-022-01649-0

[jeq270122-bib-0016] GLA . (2024). About Green Lake . Green Lake Association. http://www.greenlakeassociation.com/glaw/index.php/about‐green‐lake‐2/

[jeq270122-bib-0017] Graczyk, D. , Walker, J. , Bannerman, R. , & Rutter, T. (2010). Effects of best management practices in Eagle and Joos Valley Creeks in the Waumandee Creek Priority Watershed, Wisconsin, 1990‐2007 (Scientific Investigations Report 2010‐5119, p. 25). United States Geological Survey.

[jeq270122-bib-0018] Guo, M. (2013). Evolving bioretention techniques for urban stormwater treatment. Hydrology Current Research, 4(1), Article 1000e106. 10.4172/2157-7587.1000e106

[jeq270122-bib-0019] Hamid, A. , Bhat, S. , & Jehangir, A. (2020). Local determinants influencing stream water quality. Applied Water Science, 10, Article 24.

[jeq270122-bib-0020] Heatherly, T. , Whiles, M. , Royer, T. , & David, M. (2007). Relationships between water quality, habitat quality, and macroinvertebrate assemblages in Illinois streams. Journal of Environmental Quality, 36, 1653–1660. 10.2134/jeq2006.0521 17940265

[jeq270122-bib-0021] Hilsenhoff, W. L. (1987). An improved biotic index of organic stream pollution. Great Lakes Entomology, 20(1), 31–39. 10.22543/0090-0222.1591

[jeq270122-bib-0022] Hilsenhoff, W. L. (1988). Rapid field assessment of organic pollution with a family‐level biotic index. Journal of North American Benthological Society, 7(1), 65–68. http://www.jstor.org/stable/1467832

[jeq270122-bib-0023] Holmes, R. , Armanini, D. , & Yates, A. (2016). Effects of best management practices on ecological condition: Does location matter? Environmental Management, 57(5), 1062–1076. 10.1007/s00267-016-0662-x 26787015

[jeq270122-bib-0024] Johnson, R. (2021). Historical phosphorus flows and legacy accumulation in the watershed of Wisconsin's deepest inland lake [Master's thesis]. University of Wisconsin‐Madison. https://nelson‐public‐files.s3.amazonaws.com/green‐lake‐watershed/lake/2021+Johnson+Historical+Phosphorus+Flows+and+Legacy+Accumulation+in+Green+Lake+Watershed.pdf

[jeq270122-bib-0025] Johnson, T. , Nicekl, A. , & Evensen, E. (2011). An assessment of Hill, Roy, Silver, and Wuerches Creeks (303d Impaired Waters). Wisconsin Department of Natural Resources. https://nelson.wisc.edu/wp‐content/uploads/2011_Johnson_et_al.pdf

[jeq270122-bib-0026] Justus, B. G. , Petersen, J. , Femmer, S. , Davis, J. , & Wallace, J. (2010). A comparison of algal, macroinvertebrate, and fish assemblage indices for assessing low‐level nutrient enrichment in wadeable Ozark streams. Ecological Indicators, 10(3), 627–638. 10.1016/j.ecolind.2009.10.007

[jeq270122-bib-0027] Kiffney, P. , Richardson, J. , & Bull, J. (2003). Responses of periphyton and insects to experimental manipulation of riparian buffer width along forest streams. Journal of Applied Ecology, 40(6), 1060–1076. 10.1111/j.1365-2664.2003.00855.x

[jeq270122-bib-0029] Konopacky, J. (2017). Battling the (algae) bloom: Watershed policies and plans in Wisconsin. Environmental Affairs Law Review, 44(2), 253–329.

[jeq270122-bib-0030] Kubisch, A. , & Korff, C. (2023). Hard water. Libre Texts Chemistry. https://chem.libretexts.org/Bookshelves/Inorganic_Chemistry/Supplemental_Modules_and_Websites_(Inorganic_Chemistry)/Descriptive_Chemistry/Main_Group_Reactions/Hard_Water

[jeq270122-bib-0031] Lenat, D. R. (1993). A biotic index for the southeastern United States: Derivation and list of tolerance values, with criteria for assigning water‐quality ratings. Journal of North American Benthological Society, 12(3), 279–290. 10.2307/1467463

[jeq270122-bib-0032] Lowe, R. , & LaLiberte, G. (2017). Benthic stream algae: Distribution and structure. In G. Lamberti & F. R. Hauer (Eds.), Methods in stream ecology (Vol. 3, pp. 193–221). Academic Press.

[jeq270122-bib-0033] Minnesota Pollution Control Agency . (2017). Minnesota stormwater manual: Overview of stormwater infiltration . https://stormwater.pca.state.mn.us/index.php?title=Overview_for_infiltration

[jeq270122-bib-0034] Muenz, T. K. , Golladay, S. W. , Vellidis, G. , & Smith, L. L. (2006). Stream buffer effectiveness in an agriculturally influenced area, southwestern Georgia: Responses of water quality, macroinvertebrates, and amphibians. Journal of Environmental Quality, 35(5), 1924–1938. 10.2134/jeq2005.0456 16973634

[jeq270122-bib-0035] Oksanen, J. (2015). Multivariate analysis of ecological communities in R: Vegan tutorial . https://vegandevs.github.io/vegan/

[jeq270122-bib-0036] Oksanen, J. (2022). Vegan: an introduction to ordination . https://cran.r‐project.org/web/packages/vegan/vignettes/intro‐vegan.pdf

[jeq270122-bib-0037] Prasad, L. , Thompson, A. , Arriaga, F. , & Vadas, P. (2022). Tillage and manure effects on runoff nitrogen and phosphorus losses from frozen soils. Journal of Environmental Quality, 51(5), 978–989. 10.1002/jeq2.20396 35858102

[jeq270122-bib-0038] Prellwitz, S. , Dolan, S. , & Miner, B. (2022). Big Green Lake watershed management plan. Lake Management Planning Team.

[jeq270122-bib-0039] Pringle, C. (2003). What is hydrologic connectivity and why is it ecologically important? Hydrological Processes, 17, 2685–2689. 10.1002/hyp.5145

[jeq270122-bib-0041] SEAL Analytical . (2015a). Total Kjeldahl nitrogen‐N (copper catalyst) in drinking water, ground and surface waters, domestic and industrial wastes (AQ2 Method, EPA‐111‐A Rev. 8). United States Environmental Protection Agency. www.seal‐analytical.com

[jeq270122-bib-0042] SEAL Analytical . (2015b). Total phosphorus‐P in Kjeldahl digests (copper catalyst) of drinking water, ground and surface waters, domestic and industrial wastes (AQ2 Method, EPA‐135‐A Rev. 5). United States Environmental Protection Agency. www.seal‐analytical.com

[jeq270122-bib-0043] Stone, M. L. , Whiles, M. R. , Webber, J. A. , Williard, K. W. J. , & Reeve, J. D. (2005). Macroinvertebrate communities in agriculturally impacted Southern Illinois streams: Patterns with riparian vegetation, water quality, and in‐stream habitat quality. Journal of Environmental Quality, 34(3), 907–917. 10.2134/jeq2004.0305 15843654

[jeq270122-bib-0044] Suter, G. , & Cormier, S. (2014). Why care about aquatic insects: Uses, benefits, and services. Integrated Environmental Assessment and Management, 11(2), 188–194. 10.1002/ieam.1600 25376941

[jeq270122-bib-0045] US EPA . (1993a). Determination of total Kjeldahl nitrogen by semi‐automated colorimetry: Method 351.2, Revision 2.0. United States Environmental Protection Agency.

[jeq270122-bib-0046] US EPA . (1993b). Determination of phosphorus by semi‐automated colorimetry: Method 365.1, Revision 2.0. United States Environmental Protection Agency.

[jeq270122-bib-0047] Villeneuve, V. (2007). Documentation for the removal of Melancthon Creek from the State of Wisconsin's 303(d) list of impaired waters. Water Evaluation Section. Bureau of Watershed Management, Wisconsin Department of Natural Resources.

[jeq270122-bib-0049] Wang, L. , Brenden, T. , Seelbach, P. , Cooper, A. , Allan, D. , Clark, R. , & Wiley, M. (2008). Landscape based identification of human disturbance gradients and reference conditions for Michigan streams. Environmental Monitoring and Assessment, 141, 1–17. 10.1007/s10661-006-9510-4 17171249

[jeq270122-bib-0050] Wang, L. , Robertson, D. , & Garrison, P. (2007). Linkages between nutrients and assemblages of macroinvertebrates and fish in wadeable streams: Implication to nutrient criteria development. Environmental Management, 39(2), 194–212. 10.1007/s00267-006-0135-8 17122998

[jeq270122-bib-0052] WDNR . (2015). Diatom collections for calculation of the diatom nutrient index (DNI). WDNR Water Quality Monitoring Program.

[jeq270122-bib-0053] WDNR . (2017). Guidelines for the standard collection of macroinvertebrate samples from wadable streams v2.0. WDNR Water Quality Monitoring Program.

[jeq270122-bib-0054] WDNR . (2022). Wisconsin consolidated assessment and listing methodology (WisCALM) 2022 (Waterbody Assessment Guidance for 2021‐2022). https://dnr.wisconsin.gov/topic/SurfaceWater/WisCALM.html

[jeq270122-bib-0055] WDNR . (2024). Featured water restorations . https://dnr.wisconsin.gov/topic/SurfaceWater/FeaturedRestorations.html

[jeq270122-bib-0057] Zhao, Y. , Wang, R. , Zhang, E. , Guan, B. , & Xu, M. (2022). Aquatic ecosystem responds differently to press and pulse nutrient disturbances as revealed by a microcosm experiment. Ecology and Evolution, 12(10), 9438. 10.1002/ece3.9438 PMC9587460 36284519

