# Supplementary material for: Investigating interactions between macroinvertebrate indices, water quality parameters, and stream quality classifications in a Wisconsin agricultural watershed
Source: J Environ Qual. 2025 Dec 14;55(1):e70122. doi: 10.1002/jeq2.70122 (PMC12703226; doi:10.1002/jeq2.70122)
Supplement: Supplementary file 1 — Supplemental materials are available and include: (i) a table of the list of taxa present at each sample site, (ii) a table of the NMDS analysis results coordinates, (iii) summarized analysis from 2002 to 2022 of a larger dataset from Wisconsin's Surface Water Integrated Monitoring System database to support findings in the manuscript discussion, (iv) a table of linear regression results comparing macroinvertebrate indices with some water quality parameters from this larger dataset, and (v) four figures that visualize this supplemental analysis. [file JEQ2-55-0-s001.docx]

**Supplemental Material**

**Investigating interactions between macroinvertebrate indices, water quality parameters, and stream quality classifications in a Wisconsin agricultural watershed**

Laura M. Bates^1*^, Anita M. Thompson^1^, Laxmi R. Prasad^2^

^1^Biological Systems Engineering, University of Wisconsin-Madison, Madison, Wisconsin, USA

*Corresponding author email: [lmbates2@wisc.edu](mailto:lmbates2@wisc.edu)

^2^Agricultural and Biosystems Engineering, North Dakota State University, Fargo, North Dakota, USA

Number of supplemental pages: 7

Number of supplemental tables: 3

Number of supplemental figures: 4

The list of taxa by group from field sampling at each site in the autumn 2021 and 2022 sampling seasons are listed in Supplemental Table S1.

**Supplemental Table S1.** Macroinvertebrate taxa presence by group type, biotic index, and stream health indicator (“Excellent”, “Good”, “Fair”, or “Poor”) at each sample site during the autumn season (October-November).

| **Site^a^** | **Year** | **Group 1** | **Group 2** | **Group 3** | **Group 4** | **Index Stream Health** |
| --- | --- | --- | --- | --- | --- | --- |
| SilTribD_I | 2021 | ----- | Scud | Mussel; Fingernail clam | Midge; Leech; Snail | 1.83 (Poor) |
|  | 2022 | Caddisfly | Scud | Fingernail clam; Water mite | Snail; Sow bug | 2.67 (Good) |
| SilTribC_I | 2021 | Mayfly; Caddisfly | Scud; Cranefly; Dragonfly; Riffle beetle | ----- | Midge; Sow bug | 3.12 (Good) |
|  | 2022 | Dobsonfly | Scud | ----- | Midge; Snail; Sow bug | 3.00 (Good) |
| Roy_I | 2021 | Caddisfly | Riffle beetle; Damselfly; Scud | ----- | Snail; Leech | 3.00 (Good) |
|  | 2022 | Stonefly; Caddisfly | Scud | Fingernail clam | Snail | 3.40 (Good) |
| Dak_U | 2021 | Caddisfly | Scud; Cranefly | ----- | Snail | 3.50 (Good) |
|  | 2022 | Stonefly;  Caddisfly | Scud | ----- | Midge; Snail | 3.20 (Good) |
| SilTribB_U | 2021 | ----- | ----- | Fingernail clam | Midge; Snail | 1.67 (Poor) |
|  | 2022 | Dobsonfly; Caddisfly | Riffle beetle; Scud | ----- | Sow Bug; Snail | 3.17 (Good) |
| Spr_U | 2021 | Stonefly; Caddisfly | Scud | Crayfish; Blackfly | Midge; Snail | 3.00 (Good) |
|  | 2022 | Stonefly; Caddisfly | Scud; Damselfly | Fingernail clam | Midge; Snail | 3.14 (Good) |

**^a^Impaired sites end with “_I” and unimpaired sites end with “_U”.**

The NMDS coordinates analysis results are listed in Supplemental Table S2.

**Supplemental Table S2.** The NMDS coordinates, R^2^ value, and p-values for each water quality parameter as well as the invertebrate index score between the two sampling years across all sample sites.

| **Variable** | **MDS1** | **MDS2** | **R^2^** | **P-value** | |
| --- | --- | --- | --- | --- | --- |
| Total Phosphorus (mg/l) | 0.05074 | 0.99871 | 0.4821 | | 0.013 |
| Total Nitrogen (mg/l) | -0.14072 | -0.99005 | 0.9037 | | 0.001 |
| Dissolved Oxygen (mg/l) | -0.24121 | -0.97047 | 0.9880 | | 0.001 |
| pH | -0.00495 | -0.99999 | 0.4476 | | 0.018 |
| Transparency (cm) | -0.08641 | 0.99626 | 0.9677 | | 0.001 |
| Stream Flow (cfs) | 0.89703 | 0.44197 | 0.9981 | | 0.001 |
| Total Solids (mg/l) | 0.14743 | -0.98907 | 0.1977 | | 0.337 |
| Habitat Rating | 0.12811 | 0.99176 | 0.9002 | | 0.001 |
| Invertebrate Index | -0.99034 | -0.13869 | 0.999 | | 0.001 |

Additional environmental data from 2002 to 2022 were obtained through the WDNR Surface Water Integrated Monitoring System (SWIMS) database for Green Lake. The WDNR SWIMS database includes water quality data from multiple monitoring stations within each tributary from the last ten years, with data including macroinvertebrate indices, habitat rating, TP, TS, nitrate, and TKN. Not all water quality parameters were measured at all sites within the SWIMS database.

Linear regression, in addition to NMDS, was also used to identify associations between invertebrate indices and water quality parameters. Data from the SWIMS database were used to help understand the strength of relationships between invertebrate indices and log-transformed water quality data over a longer period (2002 to 2022). Linear regression was used to help bridge gaps in data and predict the value of invertebrate indices based on water quality parameters because not all data were collected at all sampling events per site. Water quality parameters that were not normally distributed (TP, TN, DO, and TS) as well as invertebrate indices were log transformed for this analysis. The linear regression results are displayed in Supplemental Table S2.

**Supplemental Table S3.** Linear regression results (R^2^ adjusted, F-statistic, residual standard error, degrees of freedom, and p-value) of seasonal average water quality parameters that significantly associated with average seasonal invertebrate indices across all sample sites using data from this sampling study (2021-2022) and from the WDNR SWIMS database from 2000-2022.

| **Water Quality Parameter or Year of Sampling** | **R^2^ (Adjusted)** | **F-Statistic** | **Residual S.E.** | **df** | **p-value** |
| --- | --- | --- | --- | --- | --- |
| Year | 0.05102 (0.04103) | 5.107 | 0.3559 | 95 | 0.04103 |
| pH | 0.0589 (0.04601) | 4.569 | 0.3504 | 73 | 0.03591 |
| Dissolved Oxygen | 0.1339 (0.1228) | 12.06 | 0.331 | 78 | 0.00084 |
| Total Phosphorus | 0.08636 (0.07061) | 5.482 | 0.3484 | 58 | 0.02267 |

Invertebrate indices generally increase with increasing seasonal average DO (Figure S1), pH (Figure S2) and TP (Figure S3). Invertebrate indices did not significantly associate with TN, TS, transparency, streamflow, or habitat rating in this combined seasonal average dataset.


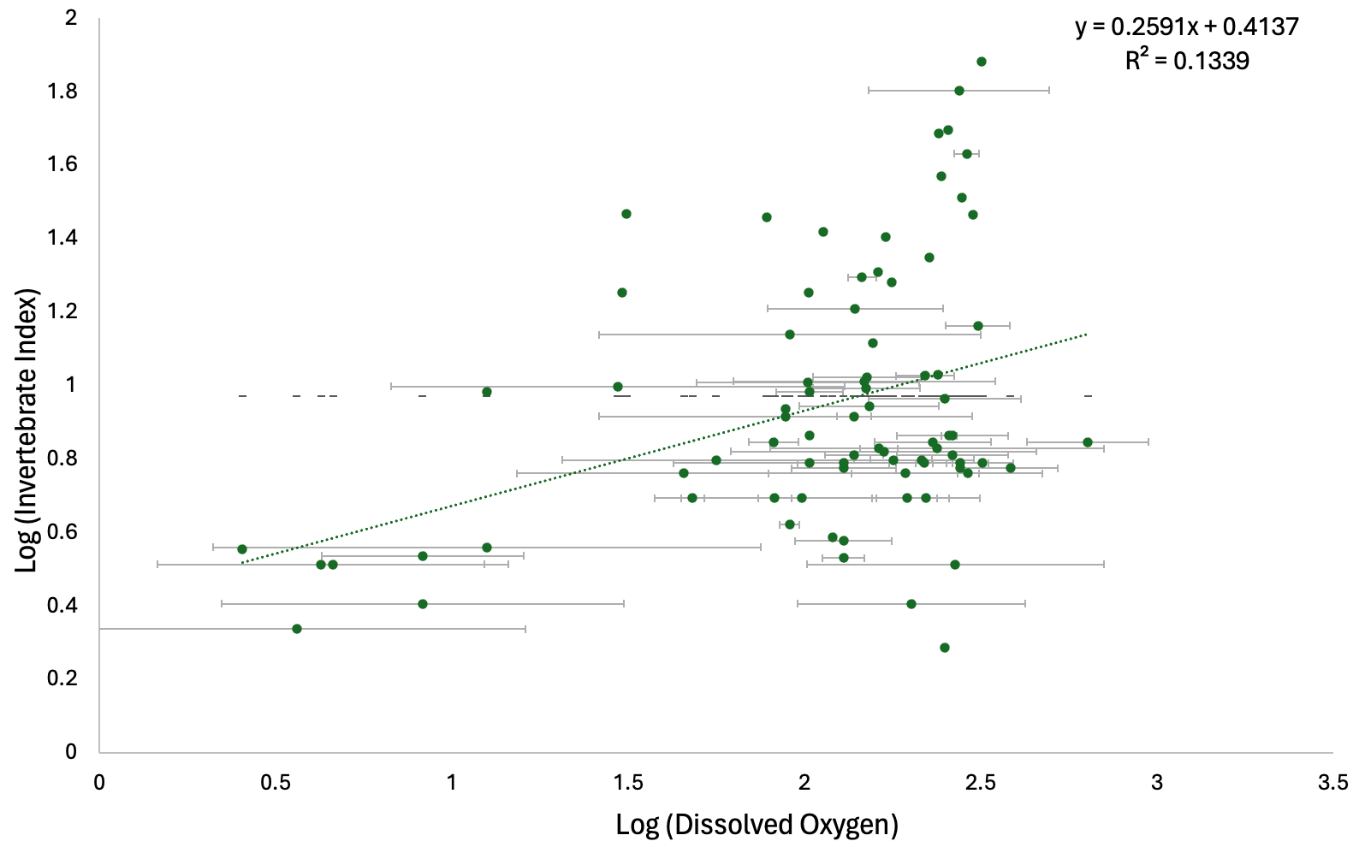


**Supplemental Figure S1** Scatter plot of the log-transformed invertebrate indices with seasonal average DO across all sample sites based on Linear Regression results (Supplemental Table S2). Error bars represent standard deviation for DO.


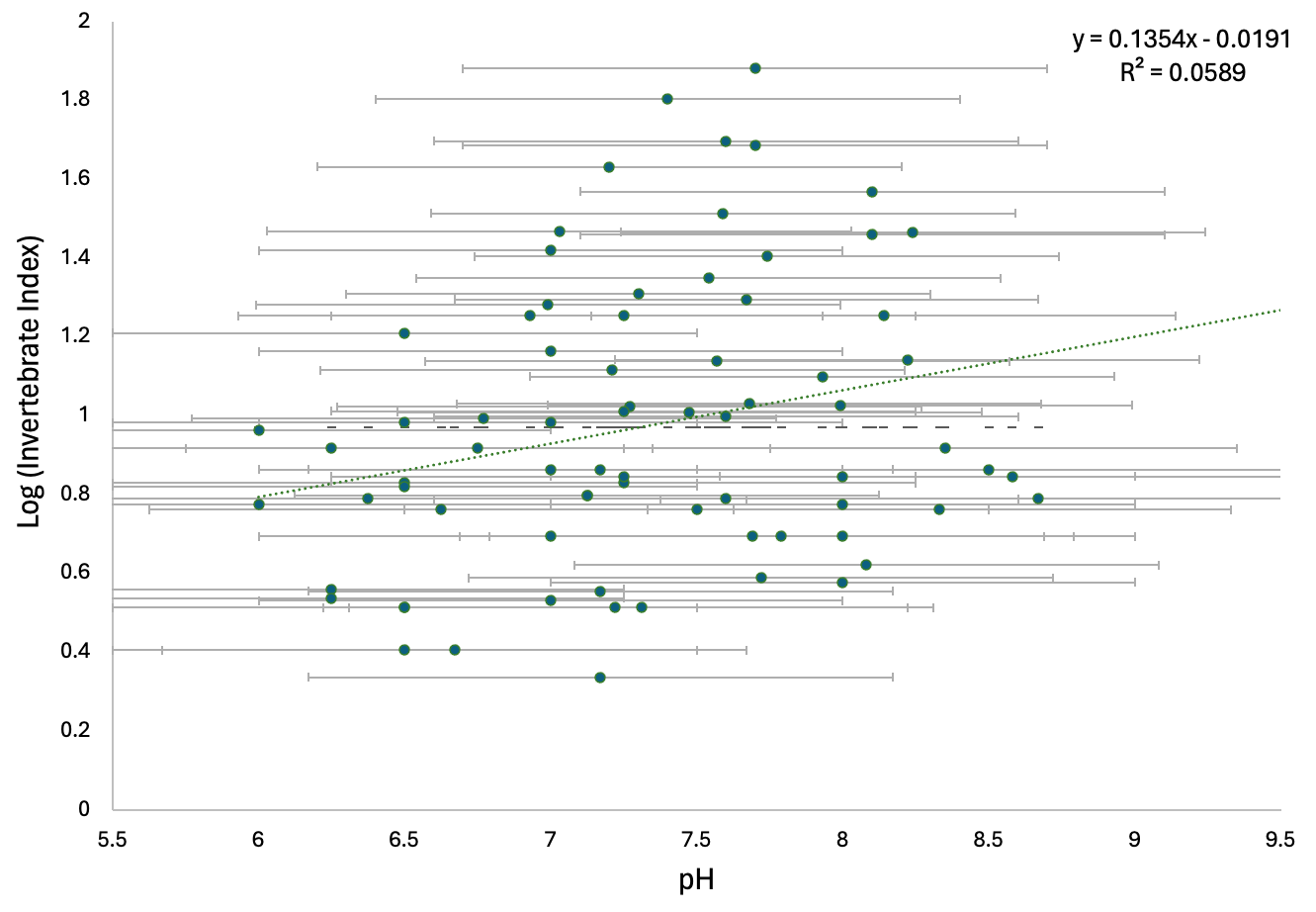


**Supplemental Figure S2** Scatter plot of the log-transformed invertebrate indices with seasonal average pH across all sample sites based on Linear Regression results (Supplemental Table S2). Error bars represent standard deviation for pH.


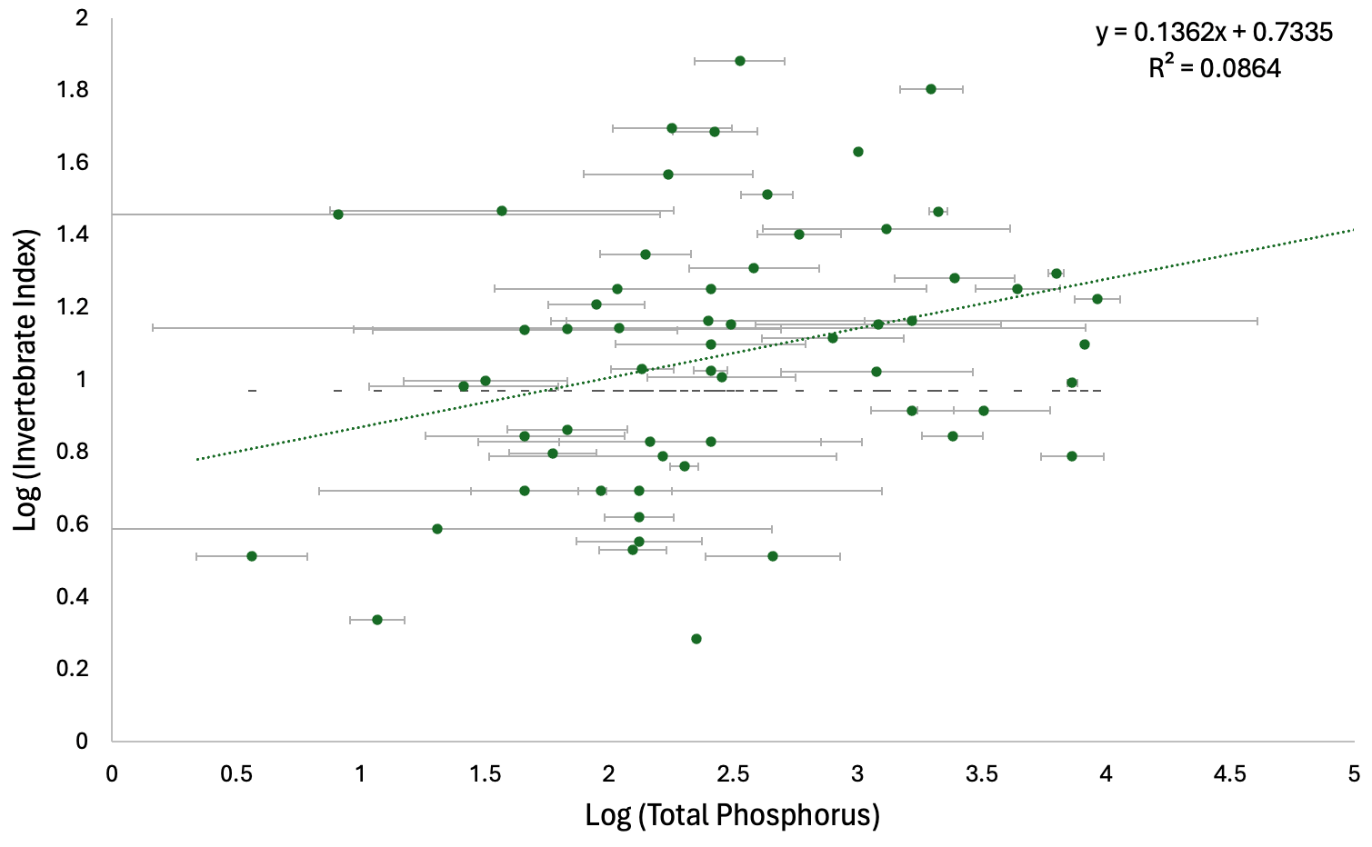


**Supplemental Figure S3** Scatter plot of log-transformed invertebrate indices with seasonal average TP at each sample site across all sample sites based on Linear Regression results (Supplemental Table S2). Error bars represent standard deviation for TP.


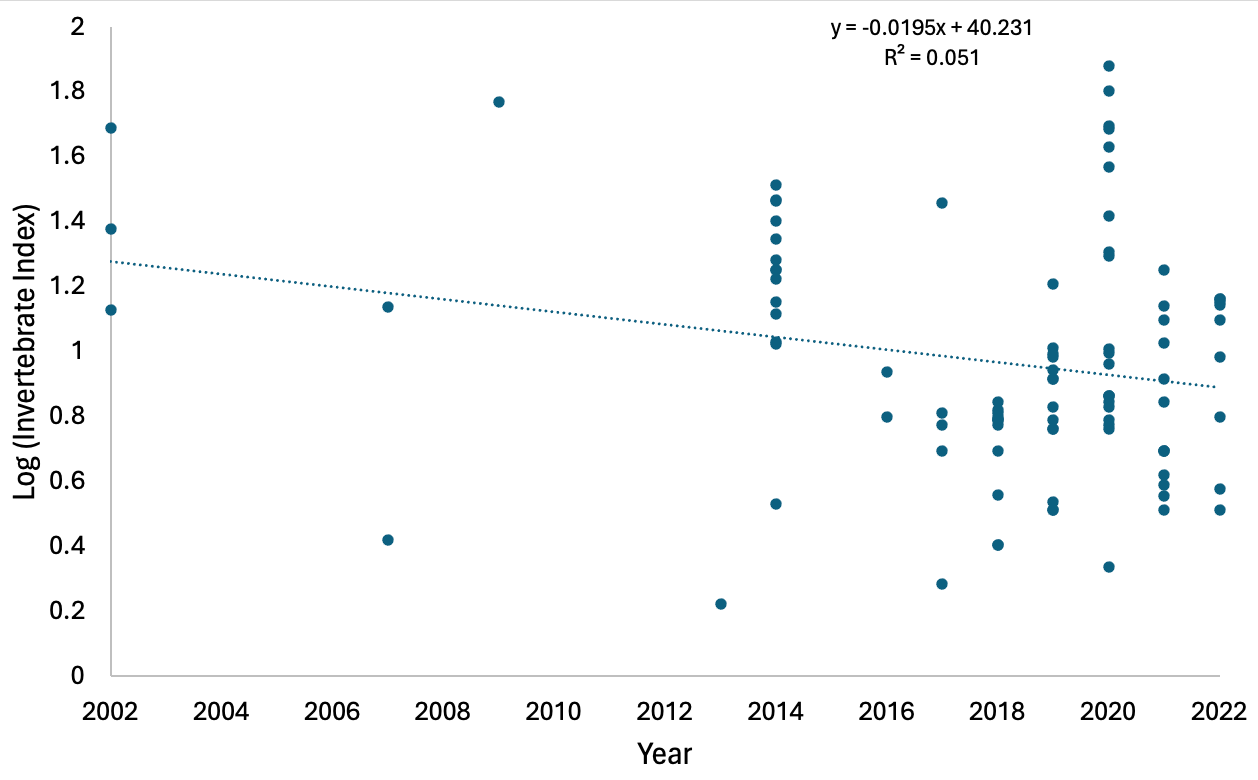


**Supplemental Figure S4** Scatterplot of invertebrate indices with sampling year across all sample sites in both sets of data (sampling data in this study and WDNR SWIMS database), based on linear regression results.

Invertebrate indices across all sites had a decreasing trend from 2002 to 2022. Sampling frequency for macroinvertebrates at streams increased dramatically in 2014, around the time that Green Lake was listed as impaired. Index scores varied widely over the past couple decades, and especially after 2010, but overall have decreased since 2002. Another limitation was that not all water quality parameters or invertebrate indices were sampled at all sites in all years in this database, or all at the same time within seasons. To mitigate this, seasonal averages of invertebrate indices and all water quality parameters are used instead of individual measurements, and linear regression was used to predict trends of invertebrate indices over time or based on water quality parameters. The null hypothesis is that there is no correlation between invertebrate indices and time, or between invertebrate indices and water quality parameters. Linear regression analysis showed a significant association between invertebrate indices and year, pH, DO, and TP (Table S2).
